# Family therapy for persons with schizophrenia: neglected yet important

**DOI:** 10.1007/s00406-022-01393-w

**Published:** 2022-03-16

**Authors:** Kurt Hahlweg, Donald H. Baucom

**Affiliations:** 1 TU Braunschweig, Schwäbische Str. 7, 10781 Berlin, Germany; 2 Psychology Department, University of North Carolina, Chapel Hill, USA

**Keywords:** Schizophrenia, Family therapy, Family intervention, Expressed emotion, Vulnerability-stress-modell, Relapse prevention

## Abstract

Since the 1950s, the observed disturbances in family relationships in which a member has been diagnosed as having schizophrenia has led many systemic family therapists to the hypothesis that these family interactions may have preceded the onset of illness and contributed to it. However, attempts at using traditional family therapy with families of patients with schizophrenia were not successful or widely adopted. With the introduction of neuroleptic medication, the treatment of schizophrenia changed dramatically, and patients often returned to their family in varying stages of partial remission, increasing the burden on relatives. Furthermore, research based on the expressed emotion concept demonstrated that the chance of relapse increases by a factor of 2.5 when a patient returns to a high-EE-family in contrast to a low-EE-family environment; consequently, the vulnerability–stress model started to guide treatment development. Based on these developments, since 1980, several psychoeducational family management programs have been evaluated showing a significant reduction in relapse when compared to standard psychiatric care from 49 to 13%. To date, at least 50 RCT studies have been published showing the effectiveness of family interventions in various culturally diverse countries. Therefore, according to the NICE and other guidelines, family intervention should be offered to all families of people with psychosis who live with or are in close contact with the patient, in conjunction with neuroleptic treatment. Despite this strong recommendation, family involvement is under-implemented in mental health care, despite its strong scientific, economic, legal and moral basis. To improve the psychosocial health of patients with psychotic disorders and their relatives, more research is necessary, as well as more training for professionals in effective family interventions.

## Introduction

Schizophrenia and other psychotic disorders are among the most severe, complex, and puzzling mental disorders and cause immense suffering for millions of people and their families worldwide. The most widely available treatment for psychosis is antipsychotic drug therapy. Randomized controlled trials (RCTs) since the 1950s have consistently shown that antipsychotic drugs effectively reduce relapses and the need for hospitalization. However, because of unsatisfactory response by some individuals, problems with adherence and disabling side effects (e.g., movement disorders, weight gain and sedation [1, 2]), the focus has begun to shift toward including psychosocial interventions such as cognitive behavioral therapy or family intervention because they “play a critical role in enhancing the patient’s overall level of functioning, quality of life, and compliance with prescribed treatments that can help reduce the risk of relapse” [3, p. 98]. In addition to cognitive behavioral therapy (CBT), family interventions also are recommended in recent clinical practice guidelines [4, 5].

The title of this paper seems simple and clear at first glance, namely: “What is the empirical status of family therapy for patients with schizophrenia?” But on a closer inspection, it becomes clear that the task is more complex. On the one hand, the term “Family Therapy” is not clearly defined, and on the other hand, there are large research gaps regarding the question of implementation and dissemination of interventions, even when the interventions are effective.

## Early history of family therapy for persons with schizophrenia

Let us begin with a brief review of the long history of family therapy which started after World War II in the USA. Influenced by psychoanalysis, some psychiatrists turned from biologically/genetically theories to environmental theories assuming that severe adult mental illness could be explained in terms of child rearing and family environment. The most influential proponents of the family etiology of schizophrenia were (main concepts in brackets) Frieda Fromm-Reichmann (1948, Schizophrenic Mother [6]), Bateson, Jackson & Haley (1956, Double Bind [7]), Lidz (1957, Marital Schism and Skew [8]), Wynne (1958, pseudomutuality [9]) and Selvini-Palazzoli (1978, Psychotic Family Games [10]).

These theories indicated that a disturbed family environment with confusing communication patterns was the major factor that drove some family members into schizophrenia. One broad principle posited that there is an issue within the family system and that one person within it becomes the designated *‘patient’* presented to services. Biologically oriented explanations were rejected and diagnostic labels avoided. Based on these theories, the field of traditional systemic family therapy rapidly grew in the 1960 s and 1970 s, regardless of the fact that none of the above-mentioned constructs received empirical support. Based on research until the present, distorted family interaction has not been found to account for the etiology of schizophrenia [11, 12].

Furthermore, the theories did not lead to effective methods of preventing or treating schizophrenia; instead, the theories often led to stigmatization of the parents as being responsible for the illness. One result of adopting these constructs was that mental health professionals either rejected family members outright or ignored their request for information or support. In a moralistic fashion, the patient’s illness was perceived to be the parents’ fault, mainly the mother’s, and, consequently, the patient had to be separated from the noxious family environment. There were little sympathy for the burden that relatives experienced.

## From hospitalization to ambulant aftercare

In the 1960s, the treatment of schizophrenia changed rather dramatically. From long-term hospitalization, often1 year or longer, patients were now treated with a relatively brief in-patient stay followed by an extended outpatient aftercare in the community. This was possible as a result of four important factors that have been identified in the literature and could become the focus of outpatient treatment [11, 12]:

### Neuroleptic treatment

Introduced in the 1950 s, neuroleptics have been shown to be effective in preventing relapse, yet even with continuous medication, approximately 27% of patients relapsed during the first year of discharge from the hospital compared with approximately 64% of patients taking placebo [2].

### Expressed emotion

The high rate of relapse stimulated research on contributing factors to this troubling pattern; in addition to medication non-compliance, a family environment high on “expressed emotion” (EE) seemed to be important in predicting relapse. Since the 1960s, levels of family expressed emotion (EE) had been found to predict relapse rates in patients with schizophrenia 9 months after hospital discharge. Expressed emotion includes two important components and is coded from the individual “Camberwell Family Interview” [13] with a relative of the patient. Ratings are based on statements made by the relative about the patient. Relatives who emit more than (a) six critical comments during the CFI and/or (b) receive a rating of three or more in the “Emotional Overinvolvement” EOI scale are categorized as high EE. Otherwise, relatives are categorized as low on EE.

Through 1990, 26 EE studies were published, and a meta-analysis by Kavanagh in 1992 [14] demonstrated that the chance of relapse increases by a factor of approximately 2.5 when a patient returns to an HEE family environment. In contrast to the 48% relapse rate among high EE families at 9 months, the relapse rate among low EE families averaged 21%; thus, the relapse rate more than doubles among high versus low EE families. Recently, O’Driscoll and colleagues [15] located *N* = 96 EE studies for their meta-analysis. They replicated the relapse findings and reported no significant effect of geographical region on global EE scores. That is, EE can be validly assessed and appears to have important implications in diverse cultures throughout the world. Furthermore, research showed that HEE is *not* correlated with patient characteristics such as the total number of symptoms at admission, aggressive behavior, or unemployment in the months before admission [12].

### Vulnerability–stress model

In 1984, the heuristic and pragmatic vulnerability–stress model was put forward by Nuechterlein and Dawson [16]. The V–S model comprises three major categories: (a) enduring vulnerability characteristics, e.g., disturbance of information processing, psychophysiological response abnormalities, and social competence deficits; (b) external environmental stressors, e.g., social stressors (negative life events, nonsupportive social network (EE attitudes); and (c) development of psychotic symptoms. It proposes that neuroleptic medication appears to be necessary to control positive symptoms, whereas psychosocial interventions seem to be indispensable for modifying unfavorable familial factors and preventing relapses.

### Burden on relatives

Patients often leave the hospital and rather than living independently, they return and live with their families in varying stages of partial remission, resulting in considerable time and effort for relatives to assist the patients. People caring for adults with schizophrenia spend an average of 6–9 h per day providing care [17, 18]. As a result, many carers are unable to work or have to take time off work to provide care. In the UK, the informal unpaid care they provide saves the National Health Service (NHS) the cost of providing comparable paid care, which is approximately £34 000 per person with schizophrenia, saving the public approximately £1.24 billion a year (based on 2012 data, [19]). While some families cope well, many have considerable difficulties fulfilling their role as caregivers. Care is usually associated with considerable stress and problems that can arise from the patients' conspicuous behavior. Their often unacceptable social behavior can lead to isolation of the family, especially the mothers. More than half of the relatives of patients with schizophrenia themselves complain of psychological symptoms requiring treatment, mostly depression and anxiety. Other negative impacts reported by the family include traumatic experiences, loss of major life goals and dreams, lack of personal and social resources, uncertainty, unpredictability, and conflict in interpersonal relationships. Furthermore, relatives are often confused, guilt ridden, and exhausted while caring for the patient [20].

Thus, while the development of new medications and the important role of family in recovery were noted through extensive research, this same accumulating evidence points out that if patients are de-institutionalized without appropriate psychosocial intervention including family or close others, there is a heightened risk for relapse and re-hospitalization.

## Family interventions

Stimulated by these changes in medication and the increasing awareness of the centrality of families in treatment, new family interventions were developed in the 1980s. Labels used to describe the interventions distinct from systemic family therapy were “Family Management, Psychoeducational Family Treatment, or Family Care”; the term “Family Therapy” was avoided.

In 1981, first results of these new interventions were published by Mike Goldstein in the ground-breaking book “New Developments in Interventions with Families of Schizophrenics” [21]. In the following years, their effectiveness to reduce relapse was further investigated in five controlled outcome studies by Anderson et al. [22], Falloon et al. [11], Goldstein et al. [23], Leff et al. [24], and Tarrier et al. [25]. In these studies, the average relapse rate in the first year for patients from HEE families receiving standard psychiatric care was approximately 50%, whereas patients with family psycho-educational treatment had a relapse rate of approximately 10%. Relapse rates after 2 years were 70% and 20%, respectively [12]. A follow-up study by Tarrier et al. [26] showed significantly fewer relapses in the family intervention group (67%) than in the high-EE control group (88%) after 8 years.

Although the individual concepts differed, there were several common components in the effective treatments that also served as a basis for future intervention efforts:

- 1) Therapists demonstrated empathy for all participants and assumed a non-pathologizing stance.
- 2) All approaches were based on the vulnerability–stress model.
- 3) The patients were on neuroleptic medication.
- 4) Patient *and* family members were included.
- 5) Intervention was relatively brief, approximately 20 sessions in the first year.
- 6) Psychoeducation on schizophrenia and neuroleptic medication was provided.
- 7) The main focus involved lowering familial stress (EE) by improving their communication and problem-solving skills.

### The Munich study: alternative neuroleptic dosage strategies and behavioral family care

Concerns about the adverse effects of neuroleptic medication, in particular the development of tardive dyskinesia, have led to the search for alternative long-term medication regimens, in particular low-dose (LDT) and targeted (= intermittent or early intervention) treatment (TMT). In LDT, patients receive about 20–40% of the usual standard dose, whereas in TMT, medication is gradually discontinued in most cases. If clinical deterioration is noted (e.g., prodromal signs occur), medication is promptly reinstated.

The Munich study was an 18-month, uncontrolled open clinical trial [27] designed to assess the relative effectiveness of LDP versus TMT, each combined with behavioral family care (BFC). Measures of psychopathology, social adjustment, side effects, family burden, expressed emotion, and relapse were assessed at baseline and periodically over an 18-month period. A significantly higher rate of relapse was observed at 18 months in patients randomized to TMT compared to those randomized to LDP (35% vs 4%). Although patients assigned to the TMT group received significantly lower mean doses of neuroleptics, there were no significant differences between the two groups with regard to side effects, global measures of social function, and overall psychopathology. Family burden was higher in TMT at 6 months, but did not differ at the 1-year and 18-month time points. However, both groups improved significantly from baseline to 12 or 18 months in almost all variables assessed. Thus, the behavioral family approach did not compensate for the problems associated with the targeted medication strategy. The results for the LDP group replicated the findings of the aforementioned Anglo-American studies [27]. The results indicate that targeted medication even in combination with BFC is not a viable alternative as a routine outpatient treatment for patients with schizophrenia [12].

### Thirty years later: current status of family intervention

Two important statistical meta-analyses were published by the Cochrane organization, *N* = 53 studies [28] and the National Institute for Health and Care Excellence NICE, *N* = 32 studies [4]; combined these two meta-analyses included all empirically validated family interventions that meet stringent inclusion criteria. In a recent German guideline published by Lincoln et al. [5], both meta-analysis and a more recent study by Camacho-Gomez and Castellvi [3] including approximately 50 RCTs were summarized. The crucial elements of family therapy identified in these reviews are clarified in the NICE guidelines noted in Table 1.

**Table 1:** Current status of family intervention (FI) vs. treatment as usual (TAU) in schizophrenia: effect sizes [5]

Outcome variable	POST	1 year FU
Total symptomatology	0.36	0.30
Relapse	0.55	0.62
Re-hospitalization	0.53	0.46
Social functioning	0.22	0.38
Cost savings (in US$)	FI > TAU	
Length of intervention (number of sessions)	0–11 < 12–51	

There was robust and consistent evidence for the efficacy of family intervention. When compared with standard care, family intervention significantly reduced (with low to moderate effect sizes): total symptomatology (0.36/0.30), rate of relapse (0.55/0.62), hospital admission (0.53/0.46), and increased social functioning (0.22/0.38) during treatment and over a 1-year follow-up period. Longer treatment achieved higher effect sizes. Economic analyses consistently reported net savings in direct and indirect costs for family interventions versus standard care.

Based on these meta-analytic findings, in 2014 NICE [4] issued the following recommendations: for people with an acute exacerbation or recurrence of schizophrenia, the following interventions should be offered:

• Oral antipsychotic medication in conjunction with psychological interventions: family intervention and/or individual CBT.

Family intervention should:

• Include the person with schizophrenia.
• Be carried out for between 3 months and 1 year.
• Take account of the relationship between the main carer and the person with schizophrenia.
• Have a specific psychoeducational component and include communication, problem-solving skills, and crisis management.

Findings consistently indicate that actively involving the *relatives* of psychotic patients will help to reduce relapse risk considerably. Furthermore, it “may help relatives better understand this disorder and its impact on personal, social and interpersonal functioning, identify exacerbated psychotic symptoms, acquire problem-solving techniques during acute episodes, and gain awareness of the importance of treatment adherence” [3, p. 106].

## Implementation and dissemination of family interventions

Despite the presence of national guidelines recommending family interventions for patients with schizophrenia, family involvement is under-implemented, under-disseminated, and under-researched in mental health care, despite its firm scientific, economic, legal, and moral basis. This appears to be the case in all industrialized Western countries [17, 29]. The inadequate implementation of family interventions may result from two important factors that are challenging for the field to address: first, there are barriers to implementing family involvement in mental health care in general. Attempting to have multiple family members present for treatment at the same time often is challenging due to scheduling, transportation, and financial constraints with family members working. Second, translating evidence-based treatments into everyday clinical practice has been difficult in the field for many years. Often therapists have been trained and treatment settings have been developed to offer treatment in one modality, and family therapy often is different from therapists’ training and how programs operate. Thus, mental health system delivery often requires strong commitment of resources and effort to provide family therapy. A reflection of these concerns and attempts to bridge the gap between scientific evidence and clinical practice guidelines have been put forward by professional institutions such as NICE [4]. “Such clinical guidelines are based on evidence synthesis from individual studies, where skilled and motivated clinicians provide an intervention to study participants, who may be carefully selected through narrow inclusion and exclusion criteria. Yet, to implement these guidelines in everyday practice, non-selected clinicians are supposed to change their clinical practice toward unselected patients and families with various comorbidities. The pathway from evidence generation to evidence synthesis and guideline development is well developed, whereas the pathway from evidence-based guidelines to evidence-based practice has more recently come to attention” [30, p. 937]. Certainly, more field (effectiveness) research is needed to investigate the reasons for under-implementation [29] of family interventions for psychosis.

One such methodologically sound study is currently underway in Norway at the Center for Medical Ethics, University of Oslo, led by Dr. Hestmark and his team [30]. Based on the established efficacy of family interventions, the investigators assume that the main benefit of family intervention for people with schizophrenia is that it may decrease the risk of relapse. It may also help people with schizophrenia consistently take their medication, make family life less burdensome and tense, and may reduce rehospitalization. “For this gain, which could be perceived as of moderate certainty, people with schizophrenia and their families should be willing to spend a significant amount of time in contact with services. We also consider it a moral imperative to involve those providing unpaid care and support, in collaboration with professional care. The deinstitutionalization of mental health care services in high-income countries has led to an increase in caring responsibilities for relatives, and their efforts are estimated to save the public health services significant costs”. [30, p. 944].

In this study, 14 Norwegian community mental health centers (CMHCs) will be involved, and 7 centers each will be randomized to TAU or family intervention. 160 patients with one family member per arm will be randomized; assessments will be at pre, 6 and 12 months; Questionnaires, interviews, register data, and health economics data will be assessed. The aims are:

- Measure the level of implementation of family interventions.
- Explore barriers and facilitators for implementing family interventions at clinical, organizational, and policy level.
- Investigate whether a higher level of implementation is associated with improved outcomes for patients and relatives.
- Analyze whether outcomes for patients, relatives, and the public health services justify the costs of implementing family interventions.

Such efforts to implement family interventions in the real world are laudatory, yet the role of the family as a major potential support system in the rehabilitation of schizophrenia is not new. Let us end with a quote from Emil Kraepelin which he published approximately 100 years ago. At that time, he already advocated early discharge of patients to their families once the most disturbing features of psychosis had diminished. He expressed his surprise that “more difficult patients behave themselves at home surprisingly well.” [31, p. 213].
